# Feasibility of ^177^Lu-PSMA Administration as Outpatient Procedure for Prostate Cancer

**DOI:** 10.2967/jnumed.124.268062

**Published:** 2024-12

**Authors:** Federico Zagni, Luigia Vetrone, Andrea Farolfi, Maria Vadalà, Elisa Lodi Rizzini, Arber Golemi, Lidia Strigari, Stefano Fanti

**Affiliations:** 1 Department of Medical Physics, IRCCS Azienda Ospedaliero, Universitaria di Bologna, Bologna, Italy;; 2 Nuclear Medicine, IRCCS, Azienda Ospedaliero, Universitaria di Bologna, Bologna, Italy;; 3 Radiation Oncology, IRCCS, Azienda Ospedaliero, Universitaria di Bologna, Bologna, Italy; and; 4 Nuclear Medicine, Alma Mater Studiorum, University of Bologna, Bologna, Italy

Targeted radionuclide therapy, as described by Goldsmith using Ehrlich’s words, is a magic bullet capable of delivering energy to a specific target to destroy it (1). Many new radiotracers have been rising, useful both for diagnosis and for therapy. Patients undergoing radiopharmaceutical therapy (RPT) become a radioactive source, requiring safety protocols for caregivers and the public. In Italy, patients may be hospitalized until the radioactive dose decays to safe levels such as in the long-practiced therapy with ^131^I (2). Similar precautions are often adopted for emerging radionuclides, resulting in the application of the same habits as for ^131^I. Instead, each case should be individually assessed to keep radiation exposure as low as reasonably achievable, balancing cost–benefit considerations. That became a necessity for [^177^Lu]Lu-PSMA-RPT, for which discharge regulations differ: some countries allow outpatient administration, whereas others require hospitalization (3). This heterogeneity impacts the worldwide diffusion of this promising cancer treatment. This editorial assesses the feasibility of [^177^Lu]Lu-PSMA administration as an outpatient procedure for prostate cancer, analyzing safety aspects, advantages, and disadvantages.

## [^177^Lu]Lu-PSMA RPT: PROCEDURES AND KINETICS

Prostate-specific membrane antigen (PSMA) is highly expressed in prostate cancer. Three PSMA imaging compounds approved by the U.S. Food and Drug Administration—[^68^Ga]Ga-PSMA-11, [^18^F]F-DCFPyL, and ^18^F-flotufolastat (Posluma; Blue Earth Diagnostics)—determine eligibility for [^177^Lu]Lu-PSMA treatment, whereas for RPT, [^177^Lu]Lu-PSMA-617 and [^177^Lu]Lu-PSMA-I&T are available, although the latter is still in clinical trials. Patients with target-positive disease can be treated successfully with RPT, achieving prolonged overall and progression-free survival (4,5). Currently in the United States, Canada, the United Kingdom, and the European Union, only [^177^Lu]Lu-PSMA-617 is approved (^177^Lu-vipivotide tetraxetan [Pluvicto; Novartis]) for PSMA-positive metastatic castration-resistant prostate cancer in patients previously treated with androgen receptor pathway inhibitors (5) and taxane-based chemotherapy. The European Medicines Agency specified for each treatment up to 6 cycles of 7.4 GBq (200 mCi) every 6 wk, with dose interruption or reduction for disease progression or unacceptable adverse events. Public exposure depends on radionuclide species, half-life, γ-emission, biokinetics, and tumor burden. [^177^Lu]Lu-PSMA has a lower external exposure rate than ^131^I for a given activity because of its lower γ-emission probability and energies. [^177^Lu]Lu-PSMA excretion takes place mostly through urine. Kinetics are modeled using a double exponential function, characterized by an initial short biologic half-life and a late, longer, half-life. Data available to date indicate an initial biologic half-life of 2–7 h, implying that about 40%–60% of the total injected activity is excreted during this time (6,7). For both [^177^Lu]Lu-PSMA-I&T and [^177^Lu]Lu-PSMA-617, strong physiologic uptake was observed in the lacrimal and salivary glands, kidneys, and small intestine on posttherapy scans, followed by medium to low uptake in the liver and spleen at all time points. The highest absorbed doses among healthy organs was in the lacrimal and parotid glands (8). Side effects included xerostomia, or dry mouth, in about 30% of patients; renal adverse events in 3%; and hematologic toxicity in only patients with extended bone disease (4). Patient-specific dosimetry appears beneficial for tailored RPT.

## ^177^LU-PSMA INTERNATIONAL SAFETY RECOMMENDATIONS

Recent reports from the International Commission on Radiological Protection (ICRP) (9,10) and the International Atomic Energy Agency, as well as guidelines from the National Council on Radiation Protection (11,12), suggest that decisions on whether to hospitalize or discharge a treated patient should be individualized on the basis of residual exposure rate and biokinetics, ensuring radiologic safety for the patient’s family and community. The patient’s physical health, psychologic condition, and ability to follow medical advice must also be considered. When treatment is being optimized, it is crucial to address radiologic safety aspects such as compliance with worker dose limits and radioactive waste management. Implementing the as-low-as-reasonably-achievable principle is key in radioprotection. The ICRP uses dose constraints as reference levels to optimize caregivers’ exposures, which can exceed public dose limits. Hospitalizing patients may reduce public exposure but poses challenges: psychologic strain on patients and families, high costs due to specialized staff and facilities, and increased exposure for health care workers. Moreover, there is a potential pool of patients who are suboptimally treated because of the waiting list relative to the long hospitalization time needed, which can be summarized as a loss of opportunity both to treat patients and to improve health care system resources. In the United States, outpatient administration allows discharge if the total effective dose to others does not exceed 5 mSv, per the Nuclear Regulatory Commission. Patients may return home 1–3 h after injection if they follow precautions such as staying adequately hydrated, using good bathroom hygiene, staying more than 0.9 m (3 ft) away from adults for 2 d and from children or pregnant individuals for 7 d, sleeping alone, and abstaining from sex for 7 d. Storing patients’ excreta and releasing them to the environment represent a long-standing debate in the scientific community, the differing approaches to which include continuous dilution, storage for decay, and environmental pathway analysis. ICRP publication 94 has reported a careful analysis of the cost–benefit ratio for ^131^I-iodide, which concludes that sewer disposal of excreta from treated patients causes exposures well within both occupational and public radiation dose limits. According to the ICRP, storing urine is not necessary since the cost is not justified by the minimal reduction in public exposure, particularly for [^177^Lu]Lu-PSMA because of its specific characteristics.

## EXPERIENCE AND DISCUSSION

Many studies are bringing RPT to earlier lines of treatment, increasing its demand. However, many health care systems are not ready yet (13,14). Because of the previous lack of demand, discussions have often been influenced by past experiences with ^131^I, despite its distinct energy characteristics compared with ^177^Lu, and by decades of cautious approaches, particularly in countries that had previously implemented more restrictive regulations. This evolving landscape encourages health care systems to reconsider the administration protocols, including whether an outpatient model would be appropriate for ^177^Lu-vipivotide tetraxetan, drawing inspiration from countries such as Italy, which have successfully adapted their practices.

RPT is safe, with very low adverse reactions, and is independently manageable by patients and care givers without needing medical intervention. This advantage allows patients to be released on the same day if they are compliant with detailed release instructions for a very few days afterward. Discharge can occur at the end of therapy, after approximately 6–8 h at the hospital for an outpatient procedure. The radiation emission immediately afterward was 17 ± 3 μSv/h measured at 1 m for 30 patients. After 6 h, this level decreased to 9 ± 3 μSv/h at 1 m. Standing 0.9 m (3 ft) away from the patient for 10 h on the day of the therapy is equivalent to undergoing 2 chest radiographs. As reported by the guidelines of the European Association of Nuclear Medicine and the Society of Nuclear Medicine and Molecular Imaging, when outpatient therapy is allowed there is the need for only 2 h of isolation to verify potential adverse events, ensure sufficient hydration, and complete the urine void. The possibility of outpatient administration leads to better compliance from patients, as hospitalization is not a psychologically comfortable experience and can disorient patients, especially elders. Simple instructions are needed: hydrate to improve radiation clearance, reducing the effects on the kidneys; continue care with the referring clinician; be careful while urinating, using a separate toilet if available or double-flushing; wash the hands with soap; keep 3 ft away from people; and sleep alone usually for 3 d. However, because adverse events are a possibility over the next few days after RPT, a trip to or a stay in the hospitable might be needed. The site needs to organize a dedicated access or pathway for patients who are treated in a day hospital setting but may have developed a complication requiring a subsequent new access to the hospital. This can be set up in either the same or a nearby hospital, depending on the local and logistic situation. An outpatient regimen is a great step forward, enabling innovative and successful therapy for prostate cancer at scale, improving patient care, and providing equitable access to the whole eligible population.

## CONCLUSION

A [^177^Lu]Lu-PSMA outpatient regimen improves the quality of life for both patients and their families, who face physical, psychologic, and social challenges. Because of the decay properties and biokinetics of [^177^Lu]Lu-PSMA, dose constraints for relatives and the public can be satisfied with few contact restrictions. An outpatient RPT regimen also reduces costs and increases the quality of service, making access to this innovation more equitable.

## DISCLOSURE

Stefano Fanti reports receiving honoraria for serving on advisory boards, giving invited presentations, and attending meetings for AAA, Astellas, Bayer, Debio, GE, IMMEDICA, Janssen, Novartis, and Telix. No other potential conflict of interest relevant to this article was reported.
